# Dynamics of non-structural carbohydrates following a full masting event reveal a role for stored starch in relation to reproduction in *Fagus crenata*

**DOI:** 10.48130/FR-2021-0018

**Published:** 2021-10-26

**Authors:** Daisuke Kabeya, Atsuhiro Iio, Yoshitaka Kakubari, Qingmin Han

**Affiliations:** 1 Department of Plant Ecology, Forestry and Forest Products Research Institute (FFPRI), 1 Matsunosato, Tsukuba, Ibaraki 305-8687, Japan; 2 Faculty of Agriculture, University of Shizuoka, Ohya 836, Shizuoka 422-8529, Japan; 3 Present address: Professor Emeritus, University of Shizuoka

**Keywords:** Allocation, growth, mast seeding, non-structural carbohydrate, resource dynamics, starch, soluble sugar

## Abstract

Although mature trees have substantial non-structural carbohydrate (NSC) storage that is well documented with respect to its capacity to buffer the asynchrony of supply and demand at the whole-plant level, its role in reproduction remains poorly understood, especially in mast seeding species. In order to elucidate whether masting depletes the whole-tree NSC storage pool, seasonal and inter-annual variations in starch and soluble sugar (SS) concentrations in branchlets, stems and coarse roots of *Fagus crenata* were measured in two stands over 5 years after a full masting event. Full masting reduced individual storage pools to 72% and 49% of the maxima in the two stands; this was observed 2−3 years after full masting. In addition, temporary reduction in starch concentration in summer due to moderate fruiting was found in roots and deep sapwood cores of stems, representing tree rings formed 20 years ago, but not in branchlets. Together with a higher starch storage pool in roots than in branchlets, these results indicate that starch stored in roots and stems is available and supports life-history traits, such as masting events, that occur irregularly. Moreover, limited rainfall in the late growing season caused a reduction in both organ NSC concentration and individual storage irrespective of masting, which further complicated the masting–NSC relationship. These findings have important implications for interpreting the role of carbon resources in masting events.

## INTRODUCTION

Non-structural carbohydrates (NSC), mainly soluble sugars (SS) and starch, provide the energy for plant growth and survival. Mature trees have substantial amounts of NSC storage^[1−3]^. At the whole-plant level, NSC buffers the asynchrony of supply and demand on temporal scales of days to years as well as across plant organs: fuelling maintenance during winter^[4]^, building leaves and supporting new growth in the spring^[5]^, and for coping with environmental stress such as drought, pathogen attacks and herbivory^[6−8]^. Compared to these functions of NSC in growth and survival, however, the role of NSC storage in reproduction is still uncertain, although it is well known that reproduction consumes substantial resources, often evaluated by carbon currency^[9]^. If storage pools are not large enough to meet demands of both growth and reproduction, depletion of the storage pool may be detected. Alternatively, a trade-off between reproduction and growth occurs within branches and/or stems^[10−13]^.

The resource depletion is expected to be particularly high in mast seeding species, i.e., perennial plants with synchronous intermittent production of large seed crops in a population^[14]^. Mast seeding or masting is a common characteristic in tropical and temperate forest trees and herbs^[14,15]^. Preferential resource allocation to reproduction during masting events has long been thought to deplete storage pools and to require more than one year's "refuelling" before the next mast event^[14,16]^. Consistently, resource depletion is a key assumption of theoretical models to explain the mechanism of mast seeding^[17,18]^. Although empirical data supporting this hypothesis has been found in orchard species^[19−21]^, the direct evidence for depletion of NSC storage pools in wild trees is mixed and species-specific, with organ-specific depletion in some species^[22,23]^, and no effects in others^[1,2]^. Therefore, the extent to which masting depletes the NSC storage pool is not clear.

Several factors may complicate assessments of the degree to which reproduction depletes NSC storage. First, the quantity of stored resources fluctuates over various time scales: daily, seasonally and inter-annually^[4,6]^. This means that it is important to select an appropriate timescale over which to measure NSC storage when assessing its relationship with reproductive events. Second, while NSC storage buffers at whole-plant level, most studies on masting-related NSC dynamics have been conducted on parts of individual organs such as branchlets^[22,24]^ or stems^[2]^ rather than whole trees^[23]^. Third, variation in the amount of NSC storage is affected by a range of factors other than reproduction events, including environmental factors such as light availability^[25]^, nutrient availability^[26,27]^ and elevation^[1]^. Therefore, long-term monitoring of both seed crops and NSC dynamics is crucial to further our understanding of the role of NSC storage in mast seeding.

In this study, we took advantage of the unique opportunity that full masting occurred in 2005 in *Fagus crenata* forests countrywide, to examine to the degree to which masting depletes NSC storage at whole tree level and to trace its replenishment processes. We measured seasonal and inter-annual fluctuations in starch and SS concentrations in branchlets, stems and coarse roots from November 2005−2009 in two stands. At the mature stand (age 83−84 years), located at 900 m elevation, no further masting events were observed after 2005, whereas moderate reproduction events occurred in the older stand (age 190−260 years) at 1500 m. Specifically, the following three questions were addressed: (1) Does masting influence temporal variation of organ NSC concentration? (2) If so, to what extent does masting deplete individual NSC storage and how long does it take to be restored? (3) From which organ does stored NSC contribute to life-history traits such as masting?

## RESULTS

### Reproduction pattern

**Figure 1:**
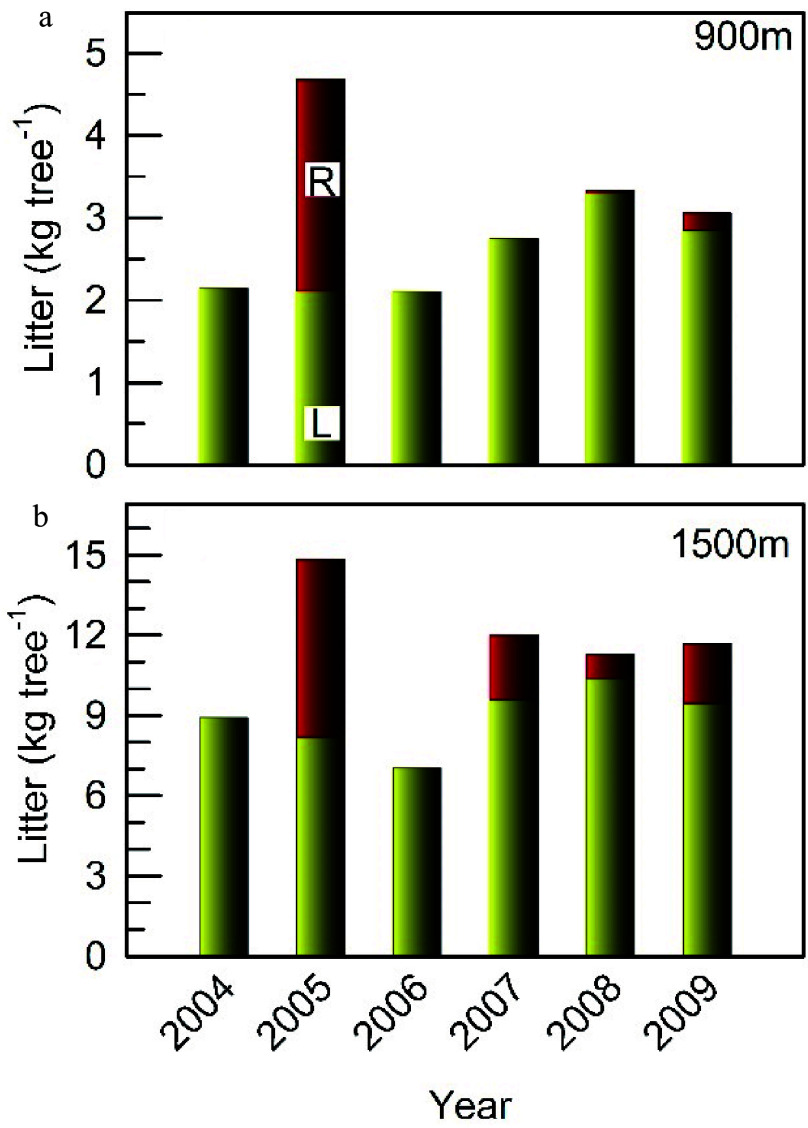
Mean dry mass of litterfall from leaves (L) and reproductive organs (R) averaged at the individual level from 2004 to 2009 at the (a) 900 m and (b) 1500 m sites (n = 5−13).

In the mature stand at the 900 m site during the 6-year monitoring period, full masting occurred only in 2005 (Fig. 1). In the older stand at the 1500 m site, additional moderate reproductive events were observed in 2007, 2008 and 2009. The amount of reproductive litter (nuts and cupules) produced in 2005 was 2.58 and 6.66 kg tree^−1^ in the mature and older stands, respectively, equivalent to the leaf litter; the quantity produced in 2007, 2008 and 2009 in the older stand, however, was only around 25%, 9% and 24% of the leaf litter respectively. Trees in the older stand that had fruited in 2005 were selected for this study, and fruited again in 2007 and 2009 but not in 2006 and 2008.

### Seasonal fluctuation

**Figure 2:**
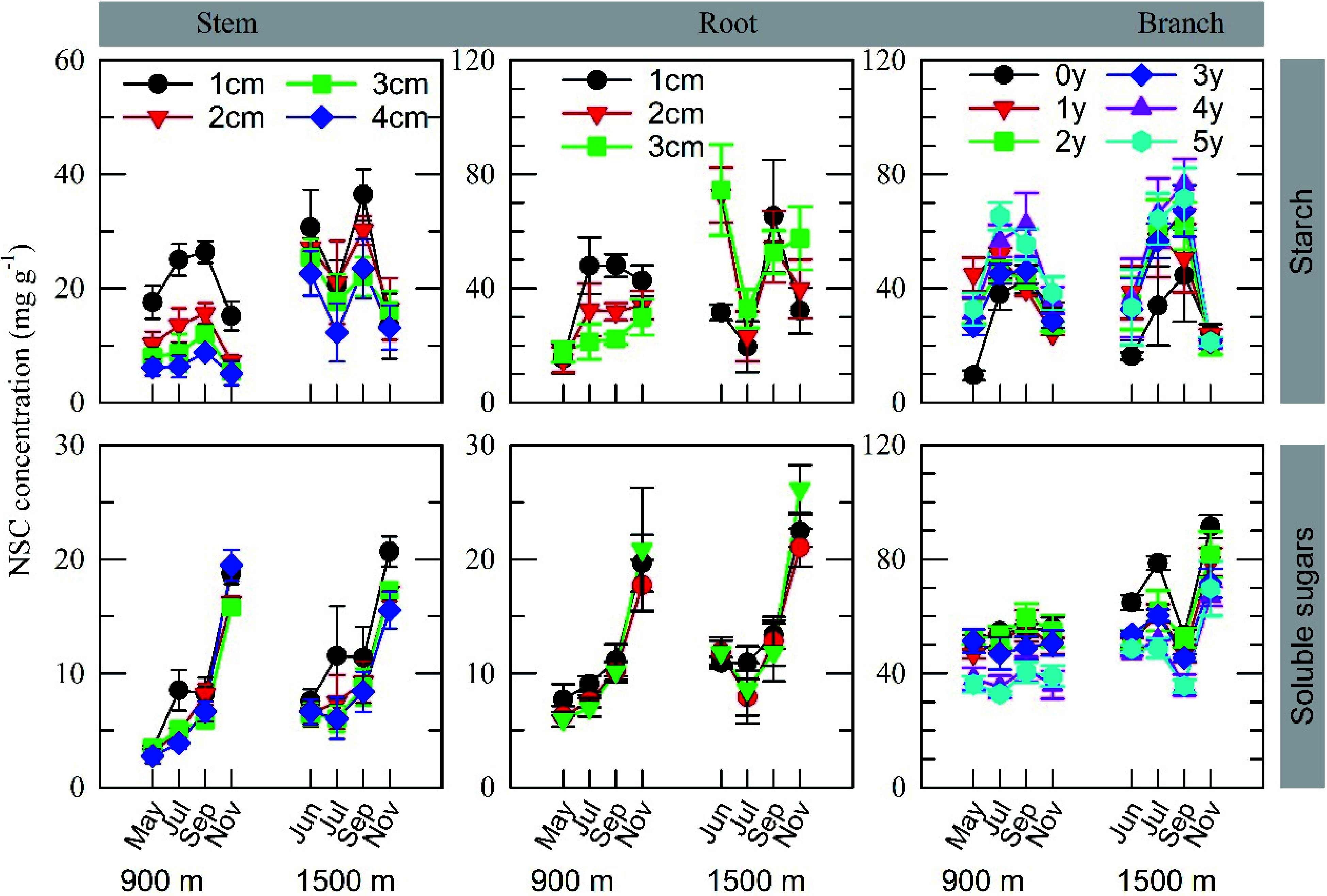
Variations in organ starch and soluble sugar concentrations for different twig ages, stem and root cores of different depths starting from the cambium at the 900 m and 1500 m sites in 2007, a year with moderate fruiting at the 1500 m site only. Values represent the mean ± SE for 3−5 individuals. The results of statistical analysis are shown in Table 1.

Both starch and SS concentration in stems and roots decreased from the outer sapwood (bark side) to the inner sapwood (pitch side) cores in both stands, except in roots from the older stand (Fig. 2 and Supplemental Fig. S1; Table 1). Starch concentration in branches increased with increasing branch age in both stands, whereas SS concentration decreased with increasing branch age.

**Table 1:** Summary statistics for the fixed effects of depth of wood cores in roots and stems and age of branches on the concentrations of starch and soluble sugars in *F*. *crenata* sampled from the 900 m and 1500 m sites. Estimated slope not equal to zero indicates significant fixed effects.

	Organ	Site elevation	Coefficients of the models			
			Intercept		Slope	
			Estimated	se	Estimated	SE
Starch	Root	1500 m	3.58	0.52	1.10	0.28
		900 m	3.77	0.34	−0.94	0.19
	Stem	1500 m	4.44	0.23	−0.73	0.07
		900 m	3.77	0.15	−0.94	0.05
	Branch	1500 m	3.07	0.13	0.25	0.04
		900 m				
Soluble sugars	Root	1500 m	1.30	0.10	−0.06	0.03
		900 m	1.20	0.09		
	Stem	1500 m	4.09	0.16	−1.02	0.04
		900 m	3.77	0.10	−0.94	0.02
	Branch	1500 m	4.98	0.31	−0.26	0.03
		900 m	4.31	0.26		
In cases where the effects of study site on the estimated slope and intercept of the model were negligible, coefficients were estimated across the sites.						

**Figure 3:**
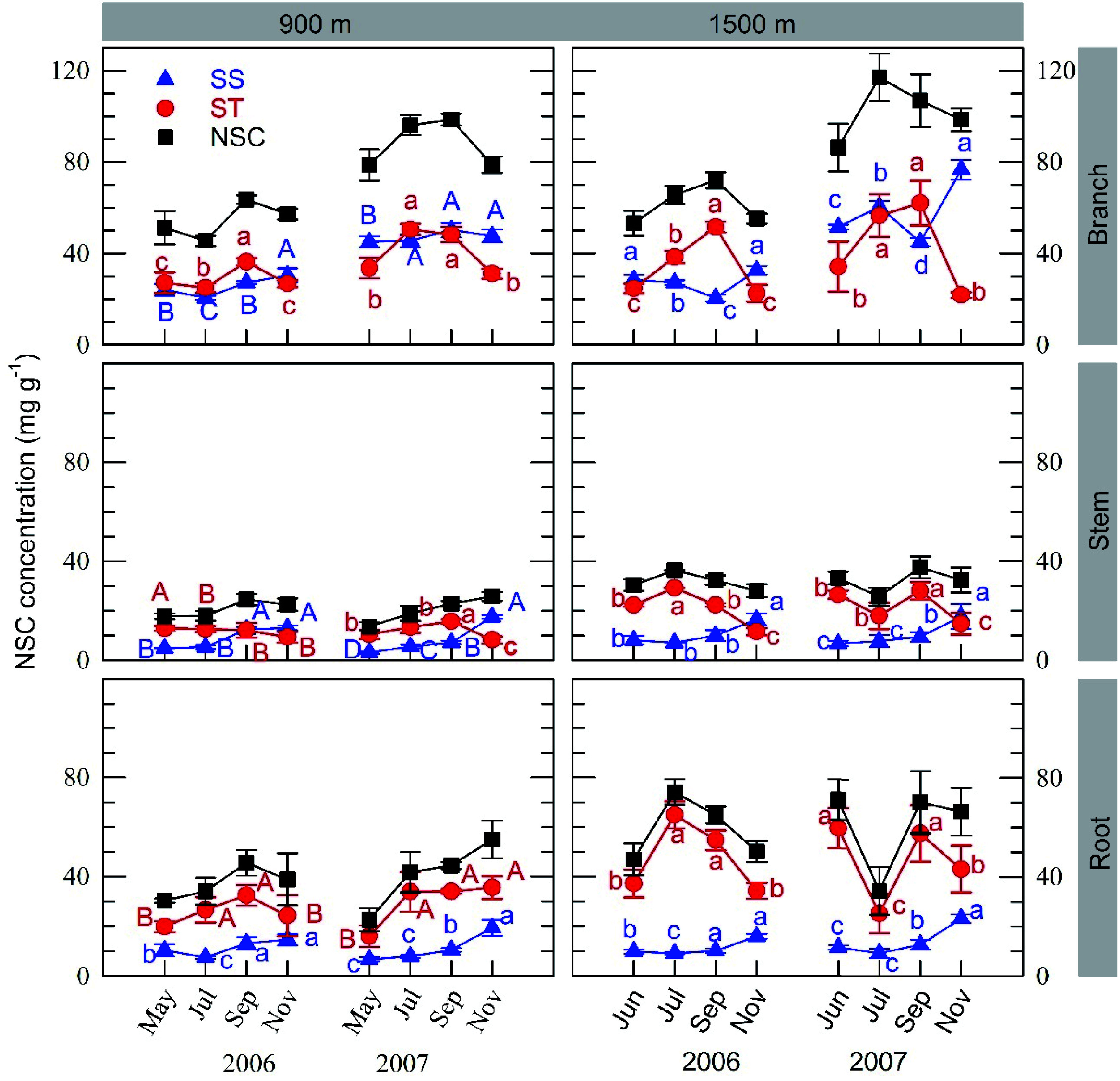
Seasonal fluctuations in organ concentrations of soluble sugars (SS), starch (ST), and the sum of both (NSC) from the branches, stems and roots at the 900 m and 1500 m sites. Values represent the mean ± SE of all branch ages or core depths for 3−5 individuals. In each graph, different capital or small letters indicate different coefficients estimated in the simplified model in Supplemental Table S2 for the 900 m and 1500 m sites, respectively. In the graphs without capital letters, parameter estimations were conducted across the two sites because the interaction terms were negligible.

The trees in both stands exhibited similar seasonal variations in starch concentration in all organs except roots and stems from the 1500 m site in 2007: it increased in summer and subsequently decreased before leaf fall (Fig. 3). This decrease was accompanied by an increase in SS concentration. At the 1500 m site in 2007 (a moderate fruiting year), however, a temporary decrease in starch concentration in summer was observed in roots and stems.

### Inter-annual variation

**Figure 4:**
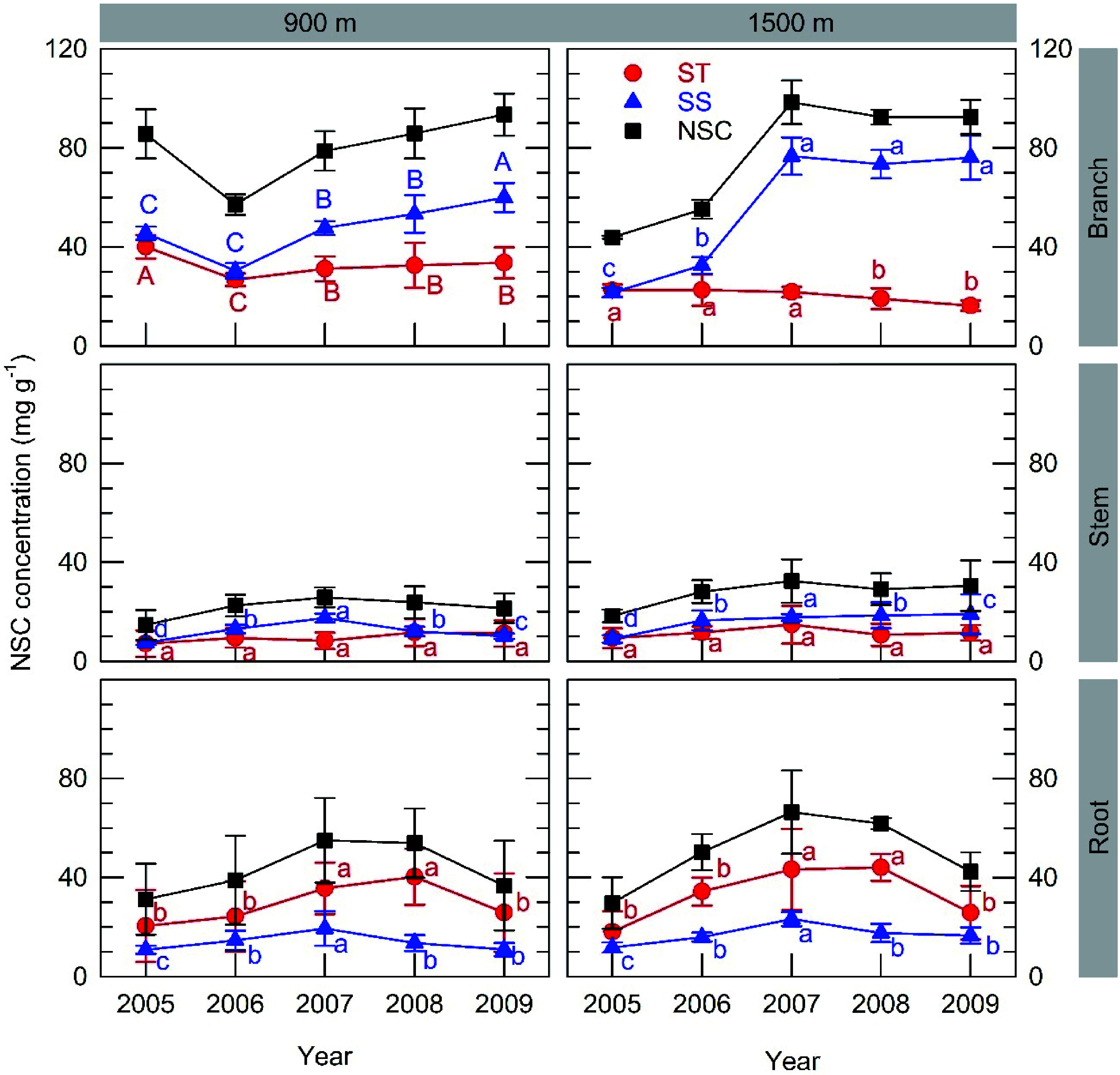
Inter-annual variations in organ concentrations of starch (ST), soluble sugar (SS) and the sum of both (NSC) after leaf fall from branches, stems and roots at the 900 m and 1500 m sites. A full masting event occurred in 2005. Values represent the mean ± SE for 3−5 individuals. In each graph, different capital or small letters indicate different values based on parameter estimations using the improved model in Supplemental Table S2 for the 900 m and 1500 m sites, respectively. Graphs that feature no capital letters present parameter estimates based on combined data from both sites because the interaction terms in these cases were negligible.

Inter-annual variations in starch concentration after leaf fall were significant in branches and roots, but not in stems (Fig. 4). Starch concentration in roots at both sites reached its lowest value after full masting in 2005, subsequently increasing to its maximum in 2007, and decreasing again in 2009 regardless of reproduction. Starch concentration in branches at the 900 m site decreased in 2006, the year following full masting and partially recovered in the following years, whereas it remained at a constant level at the 1500 m site until 2007 followed by some decreases in 2008 and 2009. The maximum starch concentration was higher in roots and branches compared to stems at each respective site.

Inter-annual variations in SS concentration were significant in all organs from both sites: decreased in 2005 due to masting and replenished up to 2007. Further increases in SS in 2008 and 2009 were observed in branches at the 900 m site but decreased in roots and stems from both sites. The maximum SS concentration was higher in branches than in stems and roots from each respective site. Comparing starch and SS concentration in the same organ, branches had higher SS concentrations, whereas roots exhibited higher starch concentrations.

### Individual NSC storage pool

**Figure 5:**
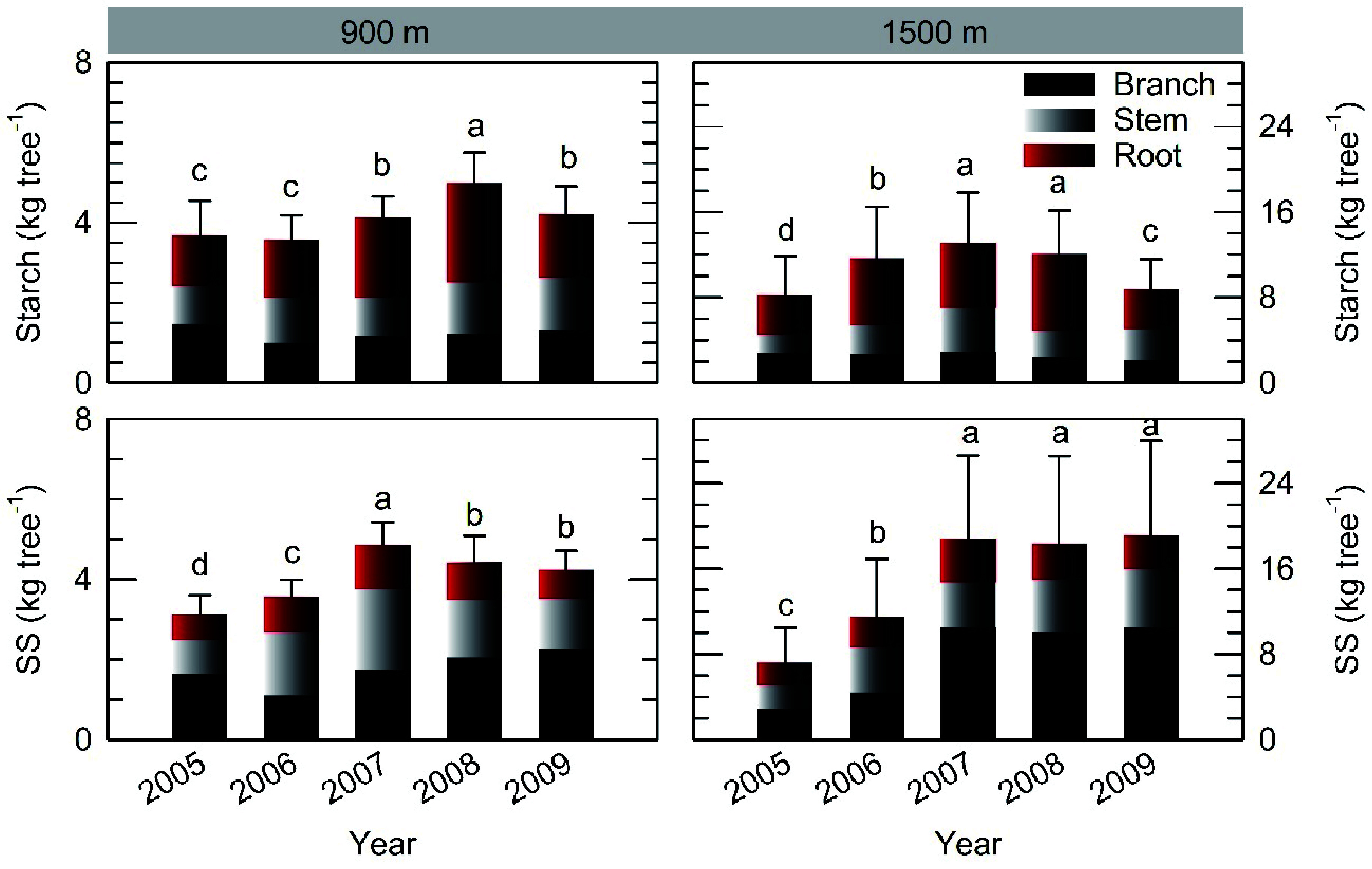
Depletion and replenishment of individual starch and soluble sugar (SS) storage pools following full masting in 2005 at the 900 m and 1500 m sites. Vertical bars indicate standard errors at tree level (n = 3−5). In each graph, different letters indicate different values based on parameter estimations using the improved model in Supplemental Table S2.

Individual starch and SS storage pools had their lowest value in 2005 after full masting at both sites, and replenished during the following 2−3 years (Fig. 5). There were some decreases in the starch pools in 2009 at both sites regardless of reproduction (Supplemental Fig. S2). Roots exhibited greater starch storage than stems and branches in both stands. In contrast, branches had larger SS pools than roots especially at the 1500 m site. The maximum NSC pool (sum of starch and SS) was 9.41 kg tree^−1^ and 31.46 kg tree^−1^ in the mature and older stands, respectively, which decreased to 72% and 49% of the respective maximum in 2005 after the full masting event.

### Annual radial stem growth

**Figure 6:**
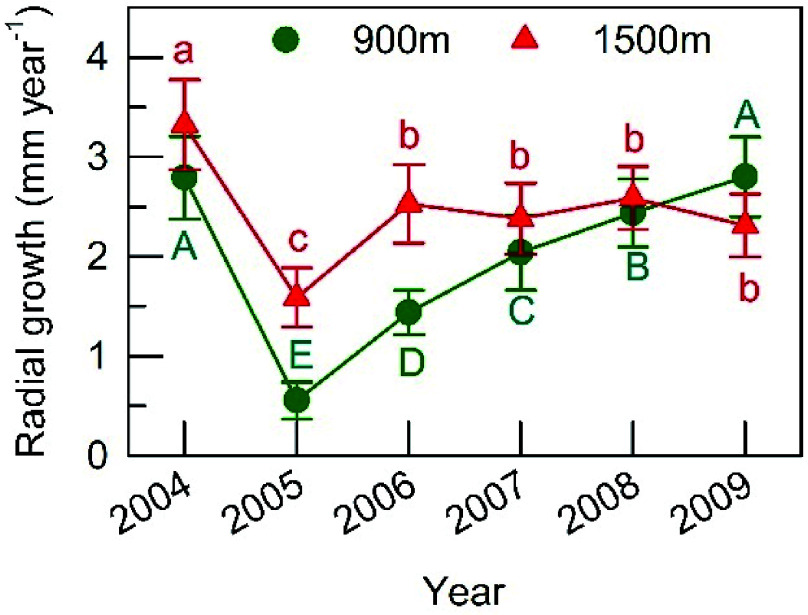
Annual radial stem increment measured at breast height in *F*. *crenata* during 2004−2009 at the 900 m and 1500 m sites. All of the sampled trees fruited heavily in 2005 (a masting year). Means and standard errors are shown. Different capital or small letters indicate different values estimated in the improved model in Supplemental Table S2.

The mean annual radial stem growth (RG) of trees that fruited in 2005 ranged from 1.6 to 3.3 mm year^−1^ at the 1500 m site (n = 17) and from 0.5 to 2.7 mm year^−1^ at the 900 m site (n = 25). At both sites, the lowest RG was recorded in the year of full masting (Fig. 6). At the 1500 m site, RG recovered very strongly in the following year and did not fluctuate much during later years. At the 900 m site, in contrast, RG increased gradually year on year after the masting event. While RG of non-fruiting trees also decreased from 2.40 ± 1.09 to 2.20 ± 0.64 mm year^−1^ at the 1500 m site (mean ± SE, n = 5), and from 2.00 ± 0.62 to 0.92 ± 0.15 mm year^−1^ at the 900 m site (n = 6) between 2004 and 2005, the magnitude of the decline between these two years was smaller than that for individuals that fruited.

## DISCUSSION

Although it has been effectively demonstrated that reproduction costs substantial resources, empirical data on depletion of stored NSC caused by mast seeding in tree species is still lacking. In *F. crenata*, seasonal variation in both SS and starch concentrations was observed in all organs, but reduction due to fruiting within the growing season was found only in starch concentration in roots and stems. Starch, unlike free sugars, is osmotically inactive and accumulates as a storage compound during times of surplus carbon supply^[4]^. Therefore, fruit burden resulted in a temporary shortage of carbon supply, suggesting that stored starch contributes to fruit growth; this also highlights the importance of timing in detecting masting-related NSC variations. In addition, the decreases in starch concentration were found in all depths of stem wood cores, which represented tree rings formed more than 20 years ago. Starch is stored in ray and axial parenchyma, and new photo-assimilates are mixed with old NSC in older rings^[28−30]^. Our results provide the first evidence that starch in deep sapwoods is available for masting events, which is consistent with it being accessible for tree growth and metabolism^[31]^. Starch stored in coarse roots and the stem plays a more important role in life-history traits (e.g. reproduction events) than local storage in branchlets, for example.

Individual NSC storage pools decreased in the year of full masting and replenishment was then observed in the next 2−3 years in both stands. These results provide evidence of a decline in NSC storage after a mast event in mature forests. However, a moderate reproduction event in 2007 did not cause reduction in either organ NSC concentration or individual storage when measured after leaf fall, at which time temporary reduction in root and stem starch concentration in summer had been refilled. These results indicate that whether a decrease in NSC storage caused by masting occurs or not depends on the quantity of fruit production. In addition, a reduction in both root starch concentration and individual starch storage was observed in 2009 irrespective of masting; this was caused by a lack of precipitation. Together, these findings have important implications for the interpretation of general masting–NSC relationships.

### Is NSC surplus not important for masting?

In this study, the maximum NSC pool in both stands had the capacity to reproduce the whole leaf crown 3−4 times, which is consistent with previous findings for ten temperate forest tree species^[2]^. After the full masting event, the individual NSC storage pool decreased to 72% (mature trees) and 49% (older trees) of its maximum level during the five-year observations. Therefore, it appears that there was still enough NSC storage to support further fruiting based on the common assumption of the "principle of allocation"^[32]^. However, this approach may overestimate the NSC storage pool that could be allocated to masting if the following factors are taken into account. First, we found that annual fine root production was about equivalent to leaf production at the 900 m site (Noguchi et al., unpublished data), which is consistent with a global review of temperate forests^[33]^. Second, the cost of reproduction may have been underestimated because "somatic costs of reproduction" were not taken into account; these may include energetic construction costs and costs associated with transport of metabolites. Third, previous meta-data analysis demonstrated that minimum NSC concentration values are as high as 46% of the seasonal maximum on average, and are similar among all types of biomes and functional types of terrestrial plants^[34]^. Taking this safety margin into account, there may not be enough NSC storage to support further fruiting after a full masting event.

Recently, ^13^C labelling of whole tree starch at the Swiss Canopy Crane (SCC) facility revealed that the seeds of three deciduous tree species were produced by current-year photo-assimilate but not by NSC storage^[35]^. The result seems to contradict this study but it is unlikely for the following reason. First, there is other evidence demonstrating that growth uses current-year photosynthate in early summer in deciduous trees, whilst maintenance respiration utilizes stored NSC^[36,37]^. Accordingly, NSC storage may contribute to respiration of developing fruits as a component of the aforementioned somatic cost of reproduction even though it could not be detected from labelled ^13^C in fruits. Second, a trade-off between annual radial stem growth and reproduction was observed in both stands, in line with previous reports^[10,11,13]^. Stem growth depression resulted from a reduction in growth rate, not the length of growing period according to a comparison of fruiting and non-fruiting trees in the same year at the 900 m site^[13]^; the stem growth finished at the end of July, as did the growth of leaves, branchlets and cupules^[38]^. These seasonal patterns of growth are consistent with temporary decreases in root and stem starch concentration in 2007, indicating contribution of starch storage in roots and stems to growth of the respective organs and fruit production at this time. In this regard, our previous study using a natural ^13^C signal at the same 900 m site indicated that stored NSC contributed to fruit development up until the middle of summer^[38]^. Third, shifting of carbon utilization for fruits from stored NSC to current photo-assimilates could occur earlier in the growing season at the SCC site compared to our study site. In this regard, the average accumulation period of carbon for seed production (using radiocarbon analyses) was estimated to be less than 1.4 years in ten temperate deciduous tree species in Japan^[39]^, providing more evidence for our interpretation. Fourth, the importance of the carbon resource in masting may differ between functional groups and may be species-specific because of differences in chemical composition of seeds and the dynamics of NSC storage. Indeed, a reduction in the interval between two masting events was observed in *Quercus crispula* based on 38 years' data^[40]^. Seeds of the genus *Quercus* are starch-rich, whereas *Fagus* seeds are nitrogen-rich. These two genera have contrasting distributions and seasonal NSC storage dynamics in their stem wood^[2,5,31,41]^.

There is additional evidence showing that nutrients such as nitrogen and phosphorous are the limiting resource for masting trees rather than carbon^[42−46]^. To acquire nutrients from soil however, carbon is indispensable as a fuel. In this regard, it is interesting that most masting trees in both temperate and tropical regions are associated with ectomycorrhizal fungal species, which enhance their nutrient acquisition at the cost of carbon resource^[47,48]^. Therefore, resource limitation in masting trees requires further research, covering the resource dynamics of masting species and the interaction between carbon-nutrient resources.

### Carbon storage organ for reproduction

Comparing the maximum concentration and individual storage pool of starch between organs during the five years of observations, roots had the highest concentration and pool in both stands. In addition, roots exhibited the maximum replenishment rate of the starch pool after full masting at the 900 m site, and in both roots and stems at the 1500 m site. These results provide more evidence that starch stored in roots and stems plays a more important role in life-history traits (e.g. reproduction) than local storage, for example, in branchlets. This is consistent with the general idea that NSC storage pools in lignified organs (coarse roots and stems) may be mobilized only in small proportions during normal functioning but can make up most of the carbon source during catastrophic events that occur irregularly^[34,49,50]^.

It is worth noting that SS in branches had the lowest value following full masting and was 2.0 and 3.6 times higher two years later at the 900 m and 1500 m sites, respectively. It has been clearly demonstrated that the SS increase in autumn mirrors the starch decrease required for winter hardening^[51,52]^. Hence, it is quite logical that SS concentrations in the branches after leaf fall were relatively higher at the 1500 m site than the 900 m site. Consequently, the masting event in 2005 may have been accomplished at the cost of SS that should have been utilized for winter hardening, and thus at the risk of cold damage. These results suggest again that carbon is also an important resource in mast seeding species although it may not be a limiting resource for floral bud initiation in *F. crenata*^[42,53]^.

### Climate factors complicate the NSC-masting relationship

A reduction in both root starch concentration and individual starch storage was observed in 2009 in both stands irrespective of masting. This was caused by a prolonged soil drought event resulting from a lack of precipitation that lasted from mid-August to early October and resulted in a dramatic decrease in leaf photosynthesis^[54]^. Suppressed annual radial stem growth did not occur in either stand because stem growth had been completed by mid-August^[13]^. Based on the seasonal variation in the organ starch concentrations in 2006 and 2007, however, less photosynthesis resulted in a reduction in starch accumulation, which agrees with previous findings, based on whole-tree ^13^C-labelling, that current-year photosynthate refills carbon storage in roots in late summer^[36]^. This also explains why the degree of temporary decrease in starch concentration was higher in roots than in stems in 2007. These results provide more evidence that starch storage in coarse roots and stems plays a key temporary role in masting events. In this way, environmental factors further complicate the detection of masting effects on resource storage^[11,55]^.

In conclusion, this study provides direct evidence that NSC storage depletion occurred in the year of masting in *F. crenata* and this depletion may be temporary, depending on the amount of seed production and environmental conditions. Starch reserved in the coarse roots and stems is available and serves as long-term carbon storage that supports life-history traits, such as reproduction and catastrophic events that occur irregularly.

## MATERIALS AND METHODS

### Study site and species

The study area was located in the Naeba Mountains in central Japan (36°53'37.9'' N, 138°46'1.5'' E), a region where natural forests are dominated by *F. crenata* over an altitudinal range from 550 to 1500 m. Eight permanent plots along the altitudinal gradient were established in 1970 for long-term ecological monitoring within the framework of the International Biological Program^[56]^. Two plots, one mature stand (age 83−84 years) at an elevation of 900 m and the other old stand (age 190−260 years) at 1500 m, were selected for this study. The general characteristics of the selected stands and trees are listed in Supplemental Table S1. The bedrock is predominantly andesite and basalt, on which a moderately water-retentive brown forest soil has formed. Further details regarding the sites have been reported by Kakubari^[56]^ and Kubota et al.^[57]^. During the period 1980–2009, mean annual precipitation and mean daily temperature at a nearby meteorological station (36°56′ N, 138°49′ E, 340 m a.s.l.; Japanese Bureau of Meteorology) were 2,221 mm and 11.5 °C, respectively. Around 3–4 m of snow accumulates during the winter. The monthly average air temperature and monthly precipitation during the growing season (May−September) during 2004–2009 generally resembled figures recorded over the preceding 30 years (Supplemental Fig. S2). However, the monthly precipitation in September 2009 was only 37 mm, which was far lower than the 30-year average for this month (185.9 mm).

### Litter-trap samples

Litter from 10−15 litter-traps placed at each elevation was collected monthly. The litter traps had a mouth of 0.25 m^2^ and were fixed about 1 m above the ground. The collected litter samples were hand sorted and material from species other than *F*. *crenata* was discarded. The *F*. *crenata* litter was separated into leaves, woody components (bark and branches) and reproductive tissues (cupules and nuts) and then dried to constant mass at 70 °C. Litter derived from bud scales and flowers was not available in some years because bud burst began when the ground was still covered with snow. These tissues were therefore excluded when estimating the annual litter production. To correct for the differences in stand density between the stands, the weight of the litter fraction in each trap was adjusted to reflect the number of individual trees in the vicinity in each study area (i.e. the weight of each fraction was divided by the stand density in the corresponding litter trap area).

### Tissue sampling

In 2005, a full masting event occurred within the *F*. *crenata* population in the study area^[53]^. Sampling began after leaf fall in November 2005. In 2006 and 2007, four sampling campaigns were undertaken in order to assess seasonal variation in the trees' NSC storage levels: one during late spring when the leaves were flushing (at the end of May for the 900 m site and at the beginning of June for the 1500 m site); one during summer when the leaves had fully matured (mid-July); one during autumn before leaf senescence had begun (late September); and one during early winter after leaf fall (November). Additional sampling after leaf fall was conducted in 2008 and 2009 in both stands to assess inter-annual fluctuations in the trees' NSC reserves. In each stand, 3−5 trees that fruited fully in 2005, located around a scaffolding tower, were selected for this study. Two 5-year old branchlets from the upper parts of each tree crown, one stem xylem core at breast height and one root xylem core exposed at the soil surface were sampled. The lengths of the stem and root cores were 4 and 3 cm, respectively, starting from the cambium. Both stem and root cores were separated into 1-cm sections and immediately frozen in liquid nitrogen in the field. Further details of the samples are presented in Han et al.^[43]^.

### Non-structural carbohydrate analyses

The NSC concentration of each organ was quantified using the method of Kabeya^[58]^. Briefly, the water-soluble fraction of each sample was extracted three times with 80% ethanol. The resulting ethanolic solutions were then combined, the ethanol was evaporated off, and the precipitate was re-dissolved in pure water. The soluble sugars in the resulting solution were quantified using the phenol-sulfuric acid method. To extract starch, the precipitate from the ethanol extractions was boiled with 0.2N KOH for 30 minutes. After neutralization with 1N CH_3_COOH, the starch in the solutions was hydrolysed into glucose using amyloglucosidase (from *Rhizopus* mold, Sigma) for 30 min at 55 °C. The glucose content of the extracts was selectively quantified using a glucose-peroxidase testing kit (glucose C-II test, Wako corp., Japan). A standard glucose solution was used as a reference for both the soluble sugars and starch content measurements.

Individual NSC storage pools were estimated by multiplying the concentration by the relevant biomass, which was estimated using allometric equations^[43]^. For stem biomass, the xylem wood fraction for which NSC analysis was conducted, not the whole sapwood proportion of the stem, was used in the estimation.

### Annual radial stem growth

Diameter at breast height (DBH) of all trees in the permanent plots at the two sites had been measured intermittently between the establishment of the plots and 2003. Since 2004, the DBH of each tree in the plot has been measured annually during the dormant season. Annual DBH data for 2004–2009 were collected and analysed. The location of DBH measurement on each tree was marked with paint to ensure that measurement was performed at the same spot each time. DBH measurements were conducted using a steel tape to measure the circumference of the stem at the marked point and then the diameter calculated based on the assumption that the stem was cylindrical.

### Statistical analyses

To characterize the relationships between the temporal patterns of the NSC concentrations in each organ and the whole-tree level, and stem growth at the different study sites, generalized linear mixed models were constructed using the SAS/STAT 9.4 software package (PROC GLIMMIX, SAS Institute, Cary, NC, USA). All of the dependent variables considered in this study were continuous, and visual inspection of a plot of the residuals against the fitted values indicated that the residuals exhibited no tendency towards dispersion. Consequently, the dependent variables, except for NSC pools at the individual level, were all assumed to be normally distributed. In individual NSC pools, log-normal distribution is more appropriate for the residual distribution. Fixed effects were evaluated using the model selection procedure described by Zuur et al.^[59]^: in each model evaluation, an appropriate variance-covariance structure was selected using the Akaike Information Criterion (AIC), which was computed using the restricted maximum log-likelihood method. ANOVA-style structure models (i.e. models containing fixed main effects and their interactions) were then constructed using the calculated AIC statistics. When effective fixed effects were identified, improved models were constructed by manually re-categorizing the parameters of the effective fixed-effects model to reduce the number of estimated parameters involved. These improved models were then used to perform multiple comparisons of the estimated parameters. AIC statistics were also computed for the improved models and used to evaluate them.

When analysing the effects of the 2005 masting event on annual stem growth, the effects of repeated measurement (i.e. measurement of the same tree in each year) were accounted for using a random intercept effect.

When analysing the seasonal and inter-annual fluctuations in the concentrations of starch and SS in each organ between the study sites, the effects of the sample fraction (sample fractions were defined in terms of depth for wood cores and age for branches), repeat sampling from individual trees, and time of sampling (season and year) on the concentrations of starch and SS in each organ were also considered; these effects were included in the models as random intercepts. Furthermore, across the sampling seasons of 2006 and 2007, the measured starch and SS concentrations were clearly related to the core depth in the root and stem samples, and to age in branches (Table 1). Therefore, when seasonal and inter-annual effects were evaluated, the carbohydrate concentrations in all branchlet age classes and all stem/root core depths were treated as independent values, and the effects of branch age and core depth of each sample were incorporated as random slope effects. After consideration of the variance-covariance structures, we finally decided to include the sampling level (i.e., each wood core and branch) random intercept effect and sampling level random slope, which adjusted the difference of core depth and branch age-class, into all models of the carbohydrate analysis.

## SUPPLEMENTARY DATA

Supplementary data to this article can be found online.
