# Supplementary material for: Dynamics of non-structural carbohydrates following a full masting event reveal a role for stored starch in relation to reproduction in Fagus crenata
Source: For Res (Fayettev). 2021 Oct 26;1:18. doi: 10.48130/FR-2021-0018 (PMC11524249; doi:10.48130/FR-2021-0018)
Supplement: Supplementary file 1 — Supplementary data to this article can be found online. [file FR-2021-0018-S1.zip › 10.48130_FR-2021-0018-Suppl-FigureS2.pdf]

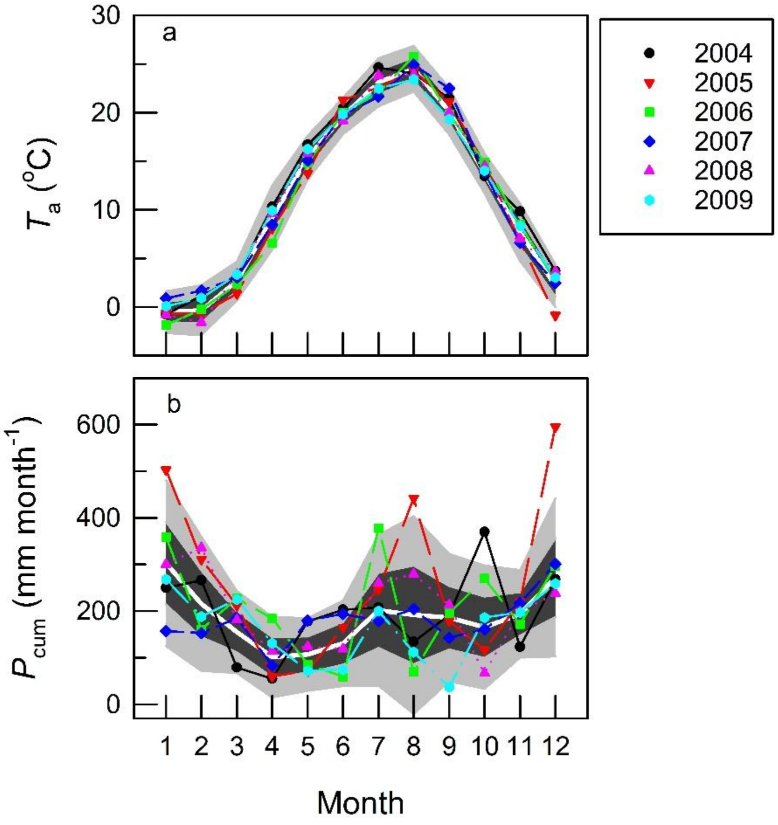

Fig. S2 Monthly mean values of selected meteorological variables recorded at an AMeDAS weather station close to the study sites in 2004-2009. White lines represent 30-year averages (1980-2010) of (a) the mean air temperature (T_a) and (b) the monthly precipitation (P_{cum}) during the growing season. Dark and light grey areas represent ± 1 SD and ± 2 SD, respectively.
