# Supplementary material for: Dynamics of non-structural carbohydrates following a full masting event reveal a role for stored starch in relation to reproduction in Fagus crenata
Source: For Res (Fayettev). 2021 Oct 26;1:18. doi: 10.48130/FR-2021-0018 (PMC11524249; doi:10.48130/FR-2021-0018)
Supplement: Supplementary file 1 — Supplementary data to this article can be found online. [file FR-2021-0018-S1.zip › 10.48130_FR-2021-0018-Suppl-TableS1.pdf]

Table S1. General information about the two stands of *Fagus crenata* on Mt. Naeba in 2005.

Elevation (m)	Slope aspect (degree)	Stand age (years)	Density (trees ha^{-1})	Tree heights (m in min.-max.)	Tree diameters at breast height (cm in min.-max.)
900	13 NE	83-84	1,190	17.2 - 22.8	20.5 - 38.1
1500	8 NE	190-260	248	19.2 - 21.6	34.1 - 62.6
