# Supplementary material for: Dynamics of non-structural carbohydrates following a full masting event reveal a role for stored starch in relation to reproduction in Fagus crenata
Source: For Res (Fayettev). 2021 Oct 26;1:18. doi: 10.48130/FR-2021-0018 (PMC11524249; doi:10.48130/FR-2021-0018)
Supplement: Supplementary file 1 — Supplementary data to this article can be found online. [file FR-2021-0018-S1.zip › 10.48130_FR-2021-0018-Suppl-TableS2.pdf]

Table S2. AICs of mixed models used to analyse the data presented in Figs. 3, 4, 5 and 6. In each analysis, the AICs of the selected ANOVA-style structure models (the model structure is noted in parentheses) and those of the improved model (see Materials and Methods) are shown together with the AICs of the null model. Bold numbers indicate the AICs of the best model in each analysis.

Figure	Substance	Organ	Year or site	AIC		
				ANOVA style model	improved model	null model
Fig. 3 (site × season)	starch	root	2006	262.6 (full)	**257.0**	281.5
			2007	366.8 (full)	**361.5**	373.8
		stem	2006	175.4 (full)	**171.5**	218.9
			2007	248.6 (site + season)	**246.6**	258.8
		branch	2006	360.5 (season)	**357.5**	371.2
			2007	638.2 (season)	**634.5**	659.6
	sugars	root	2006	8.8 (season)	**6.8**	77.9
			2007	51.2 (site + season)	**49.8**	151.1
		stem	2006	83.5 (full)	**76.3**	144.2
			2007	1.29 (full)	**-0.01**	99.8
		branch	2006	217 (full)	**213.2**	301.3
			2007	520.7 (full)	**517.5**	604.1
Fig. 4 (site × year)	starch	root		367.6 (site + year)	**363.1**	430.6
		stem		**205.2** (site)	-	254.3
		branch		566.7 (full)	**559.0**	801.5
	sugar	root		87.5 (site + year)	**84.5**	202.6
		stem		97.9 (year)	**96.1**	226.5
		branch		1036.1 (full)	**1030.8**	1200.1
Fig. 5 (year)	starch		900m	3.4	**-0.36**	14.0
			1500m	-6.4	**-6.90**	13.3
	sugar		900m	-21.1	**-22.92**	9.6
			1500m	-15.0	**-17.33**	30.5
Fig 6 (site × year)	-	-	-	-359.1 (full)	**-366.2**	-270.9
