# Supplementary material for: Dynamics of non-structural carbohydrates following a full masting event reveal a role for stored starch in relation to reproduction in Fagus crenata
Source: For Res (Fayettev). 2021 Oct 26;1:18. doi: 10.48130/FR-2021-0018 (PMC11524249; doi:10.48130/FR-2021-0018)
Supplement: Supplementary file 1 — Supplementary data to this article can be found online. [file FR-2021-0018-S1.zip › 10.48130_FR-2021-0018-Suppl-FigureS1.pdf]

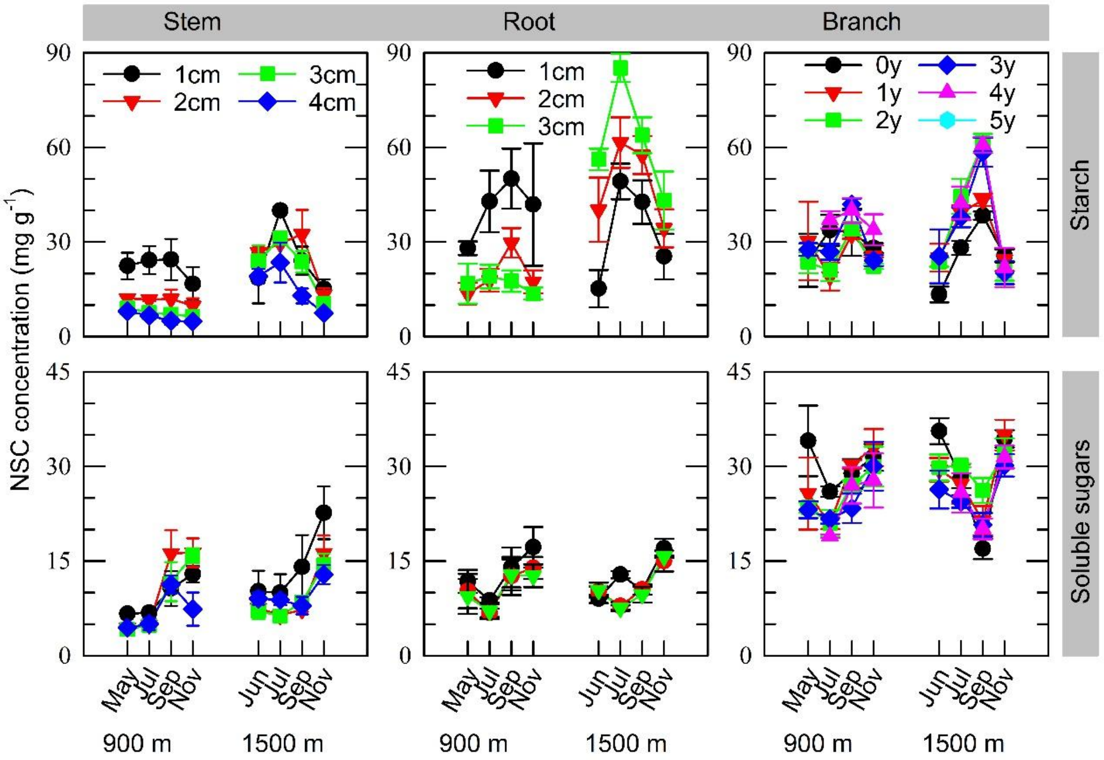


**Fig. S1** Variations in organ starch and soluble sugar concentrations of different twig ages, stem and root cores of different depths starting from the cambium in a younger stand at 900 m elevation and old stand at 1500m elevation in 2006, a year without a masting event. Values shown are mean ± SE for three to five individuals. The results of the model selection process are shown in Table 2.
